# Therapeutic potential of orally applied KB-R7943 in streptozotocin-induced neuropathy in rats

**DOI:** 10.1016/j.heliyon.2024.e27367

**Published:** 2024-03-12

**Authors:** Pavlina Andreeva-Gateva, Milen Hristov, Margarita Strokova-Stoilova, Natasha Ivanova, Zafer Sabit, Slavina Surcheva, Mihail Beliakov, Georgi Karakashev, Ivan Sukhov, Daria Belinskaya, Natalia Shestakova

**Affiliations:** a Department of Pharmacology and Toxicology, Faculty of Medicine, Medical University of Sofia, Bulgaria; b Institute of Neurobiology, BAS, Bulgaria; c Department of Pathophysiology, Faculty of Medicine, Medical University of Sofia, Bulgaria; d Laboratory of Chemical Analytical Control and Biotesting, Research Institute of Hygiene, Occupational Pathology and Human Ecology, St Petersburg, Russia; e Sechenov Institute of Evolutionary Physiology and Biochemistry, Russian Academy of Sciences, St Petersburg, Russia

**Keywords:** Diabetic neuropathy, KB-R7943, Amitriptyline, Behavior tests, Pharmacokinetics

## Abstract

Both peripheral neuropathy and depression can be viewed as neurodegeneration's consequences of diabetes, at least in part coexisting with or resulting from sodium-calcium dysbalance. This study aims to assess the therapeutic potential of the orally applied reverse-mode inhibitor of the sodium-calcium exchanger (NCX) KB-R7943 in the streptozotocin (STZ) diabetes model in rats. A pilot pharmacokinetic (PK) study with high-performance liquid chromatography with high-resolution tandem mass spectrometric detection revealed higher drug exposure (AUC), lower volume of distribution (Vd) and clearance (Cl), and faster decline of the plasma concentration (ƛ) in rats with diabetes vs. controls. Brain and heart accumulation and urinary excretion of the unmetabolized KB-R7943 at least 24 h were also demonstrated in all rats. However, heart and hippocampus KB-R7943 penetration (AUC_tissue_/AUC_plasma_) was higher in controls vs. diabetic rats. The development of thermal, mechanical, and chemical-induced allodynia was assessed with the Cold plate test (CPT), Randall-Stiletto (R–S) test, and 0.5% formalin test (FT). Amitriptyline 10 mg/kg, KB-R7943 5 mg/kg, or 10 mg/kg p.o once daily was applied from the 28th to the 49th day. The body weight, coat status, CPT, R–S, and FT were evaluated on days (−5), 0, and 42. On day 41, a forced swim test and 24-h spontaneous physical activities were assessed. The chronic treatment effects were calculated as % of the maximum. A dose-depended amelioration of neuropathic and depression-like effects was demonstrated. The oral application of KB-R7943 for potentially treating neurodegenerative consequences of diabetes merits further studies. The brain, heart, and kidneys are essential contributors to the PKs of this drug, and their safety involvement needs to be further characterized.

## Highlights

• KB-R7943 demonstrates higher drug exposure and lower volume of distribution and clearance in rats with diabetes vs. controls.
• Heart and hippocampus KB-R7943 penetration is higher in controls.
• Oral doses of 10 mg/kg of KB-R7943 and amitriptyline have comparable antinociceptive effects in STZ-induced neurapaty.

## 1 Introduction

Diabetic neuropathy (DN) is a progressive degeneration of peripheral nerves, affecting between 6 and 51% of adult patients, depending on age, duration of the disease, and glycemic control [1]. It is primarily a sensory neuropathy with late-stage involvement of motor nerve dysfunction. Autonomic axons are also targeted. Progressive diabetic neuropathy involves retraction and ‘dying back’ of terminal sensory axons in the periphery, with relative preservation of the perikarya (cell bodies) that reside in the dorsal root ganglia being debated [2]. Researchers focused the last several decades' efforts on investigating metabolic and redox-state modulators as putative DN treatment modalities, e.g., aldose-reductase, protein kinase C, or advanced glycation end-product inhibitors.

Additionally, suggested treatment options as antioxidants failed to prove efficacy, except alfa-lipoic acid, used in some parts of the world for DN. Tricyclic antidepressants, serotonin-norepinephrine reuptake inhibitors, and gabapentenoids are the mainstay in the management of painful DN; however, the treatment goal is challenging to reach [3]. A disease-modifying treatment for this condition does not exist.

The vulnerability of peripheral nerves in DN is partly explained by the abundant neuronal expression of ion channels and insufficient energy supply for proper functioning in a diabetic state [4]. Calcium dyshomeostasis is thought to play an essential role in DN. An increased intraaxonal Ca2+ overload was demonstrated in experimental diabetes, with subsequent impairment of the axonal mitochondrial function and apoptosis [5]. Ca2+ enters the axon by various Ca2+ channels and sometimes by the Na+/Ca2+ exchangers (NCX), but the leading active extruders of Ca2+ from the cell are NCX [6]. From the three known isoforms of the NCX, isoforms NCX2 and NCX 3 are highly expressed in neuronal tissue, both in peripheral and central neurons, extruding Ca2+ in exchange for Na+. However, NCX can operate in both a foreword and reverse mode depending on the electrostatic and electrochemical gradient.

In DN, several factors contribute to the reversal of the NCX-electrolyte transport with resulting calcium overload; e.g., hyperglycemia-induced NCX activity was strongly associated with the NCX reverse mode [8]. Transient glucose deprivation without hypoxia can occur in diabetes with insulin resistance [9]. As a result, Na + influx reverses both K-dependent (NCKX) and K-independent (NCX) exchangers and contributes to NMDA cytotoxicity [10]. On the other hand, hyponatremia, a common complication of diabetes [11], would decrease the sodium gradient and reverse the direction of NCX [12]. Directly diabetes-induced modifications of the exchanger molecules in DN (by the mitochondrial alteration with subsequent oxidative stress, endoplasmatic reticulum stress with misfolded protein formation [13], Cajal body dysfunction with spliceosome alterations [14], epigenetics [15], or posttranslational protein modifications [16]) can also be anticipated.

Depression is a common comorbidity of the DN. Its prevalence ranges from 20 to 25% of those suffering from painful DN [17] to nearly half of the patients living with diabetic foot ulcers [18]. Many are known, and much more is unknown about the pathogenesis of depressive disorders. However, one of the mechanistic approaches goes toward studying dysregulations of electrolytes in the brain. The fundamental roles of Ca2+ in neuronal excitation, transmitter synthesis and release, and synaptic function and plasticity have been under investigation for almost 100 years [19]. Calcium ions are involved in the presynaptic release of neurotransmitters and directly and indirectly modulate neuronal excitability, excitotoxicity, long-term potentiation, and long-term depression [20].

KB-R7943 is a substance identified as a nonspecific blocker of the NCX reverse mode [21]. It is a three-fold more potent inhibitor of NCX3 than NCX1 and NCX2 [22]. It also blocks K+ channels, Na+ and Ca2+ channels [23], TRPC3, -6, and -7 [24], NMDA receptors, and mitochondrial complex I [25], making it not that selective. KB-R7943 was found to suppress brain ischemia-reperfusion-induced injury [7], seizures [26], or acting against neuropathic pain [27]. However, testing KB-R7943 in the DN model is still a challenge. This study aimed to investigate the tissue distribution and neuropharmacological effects of the reverse-mode inhibitor of the sodium-calcium exchanger (NCX) KB-R7943 in the diabetic neuropathy (DN) model. Amitriptyline was chosen as a comparator, firstly, as a standard treatment option for diabetic neuropathy; secondly, as a well-known antidepressant drug] and thirdly, based on the discovered potential mechanism to inhibit NCX, among others [28].

## 2 Material and methods

### 2.1 In silico evaluation of physicochemical property, pharmacokinetics, and toxicity

For planning our in vivo evaluations, the following in silico tools were utilized: ADMETlab 2.0 and ProTox-II. Data about physicochemical properties, pharmacokinetics, and toxicity are essential to choosing an appropriate route of administration, deeming that for chronic use of this substance, oral will be more convenient than daily i.p. application. Although the in silico approach in toxicology is not regulatory approved [29], it is essential to be aware of some toxicity clues of this relatively new test compound. ADMETlab 2.0 is an integrated online platform for predicting of absorption, distribution, metabolism, excretion, and toxicity, known together as ADMET properties. ProTox-II is entirely focused on toxicity prediction. Chemaxone software was used for liposolubility assessment as LogD and hydrophilic-lipophilic balance number (HLB). A generic physiologically based kinetic (PBK) model for rats (QIVIVE) was used to predict plasma protein binding and tissue/plasma distribution by the Rodger and Rowland method [30].

### 2.2 Drugs and chemicals

**Fig. 1:**
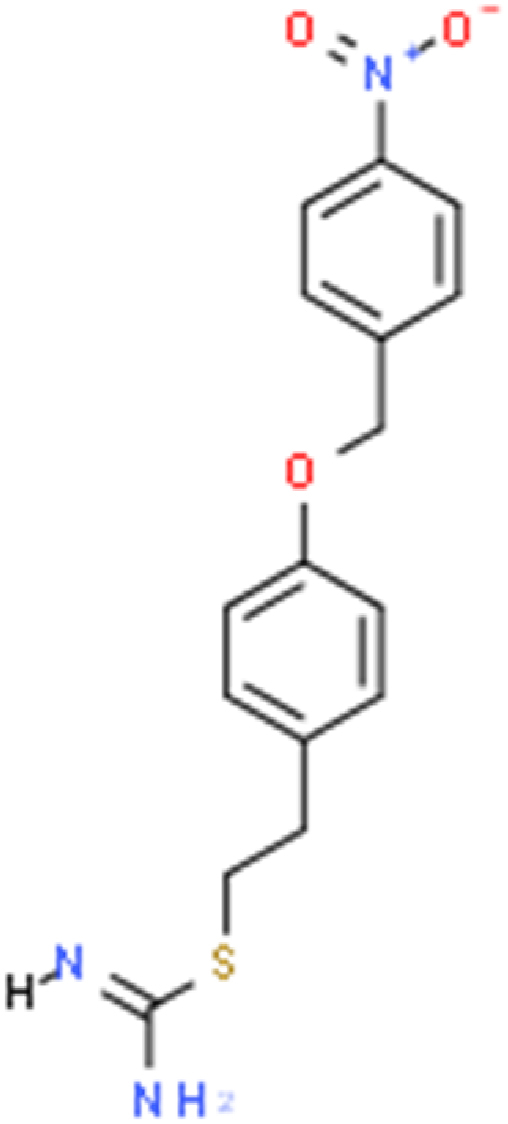
KB-R7943 [(2-{4-[(4-nitrophenyl)methoxy]phenyl}ethyl)sulfanyl]methanimidamide.

KB-R7943 is an isothiourea derivative, quaternary ammonium-containing molecule, with a molecular weight 372, logP 3,83, logD from 1,41 to 1,52 (for pH from 1,7 to 8,0), HLB 16,31 pKa 10 (data from Chemaxone) (Fig. 1).

KB-R7943 was from Tocris Bioscience (Bristol, UK). STZ, citrate buffer, and amitriptyline were from Merck KGaA (Darmstadt, Germany), and OraPlus from Perrigo (Dublin, Ireland). The formalin test used a freshly prepared 0.5% formaldehyde solution in saline. Amitriptyline (purity not less than 98%) was obtained from Merck (USA), аcetonitrile (ACN, HPLC grade) manufactured by ITW Group (USA), hexane was obtained from Cryochrom (Russia), formic acid produced by Sigma-Aldrich (USA), purified water was prepared in the laboratory with a Milli-Q system from Millipore (USA), ethylacetate (purity not less than 99,8%) produced by Vekton (Russia), reserpine (99%, HPLC grade) manufactured by “Sigma-Aldrich” (USA) was used as an internal standard.

### 2.3 Equipment

Analytes were extracted and analyzed from the matrix samples using the following equipment: ultrasonic unit Elmasonic S30 (Elma-Hans Schmidbauer GmbH, Germany), rotatory shaker Heidolph (Heidolph instruments GmbH, Germany), laboratory centrifuge Eppendorf miniSpin plus (Eppendorf, Germany), chromatography columns Agilent Zorbax SB-C8 150 mm × 4.6 mm × 1.8 μm, Zorbax SB-C18 150 mm × 4.6 mm × 1, 8 μm, and Zorbax SB-C18 250 mm × 4.6 mm × 1.8 μm. Extracts’ concentration was performed using an FMS Super Vap 24 sample preparation system (FMS Inc., USA). The analysis of the sample extracts was conducted with Dionex UltiMate 3000 (Thermo Scientific) with Q Exactive detector (Thermo Scientific) with electrospray ionization (ESI) and liquid chromatograph, LC-20, equipped with an autosampler and mass-selective detector LCMS-8050 with electrospray ionization at atmospheric pressure, (Shimadzu, Japan).

### 2.4 Liquid chromatography-tandem mass spectrometry (LC–MS/MS)

Chromatographic separation was achieved by injecting 5 μL of the sample extract (on each instrument) into a Zorbax SB-C8 150 mm × 4.6 mm × 1.8 μm column. The mobile phase was a mixture in a gradient mode of two components: solvent A - 0.1% formic acid in water and solvent B - 0.1% formic acid in acetonitrile. The flow rate was set to 0.400 ml/min with the following gradient program: 0.0–0.5 min 90% solvent A, 0.5–10.0 min 10% solvent A, 10.0–12.0 min 10% solvent A, 12.0–12.1 min 90% solvent A, 12.1–13.0 min 90% solvent A. Mass spectrometric detection was performed with positive electrospray (ESI(+)). Analytes were determined by selecting two characteristic target reactions (MRM transitions) and the retention time of the analytes. The following MS parameters were kept constant during the analysis: nebulizer voltage 4800 V for positive ionization. The temperature of the cone was set at 300 °C, the temperature of the heated probe at 400 °C, the gas flowed through the nebulizer at 3 l/min, and the flow rate of the drying gas was 10 l/min. Two product ions and one precursor ion were selected for analyte identification (Table 1).

**Table 1:** CAS number, molecular weight (MW), retention time (t_R_), multiple reaction monitoring (MRM) 1 and 2 employed for identification and quantification of the compounds and internal standard (IS).

Compound	CAS number	MW	t_R_ (min)	MRM1	MRM2
KB-R7943	182,004-65-5	427.5	10.28	332.11 > 256.15	332.11 > 256.15
Rezerpin (IS)	50-55-5	608.7	10.96	609.28 > 195.10	609.28 > 174.05

**Fig. 2:**
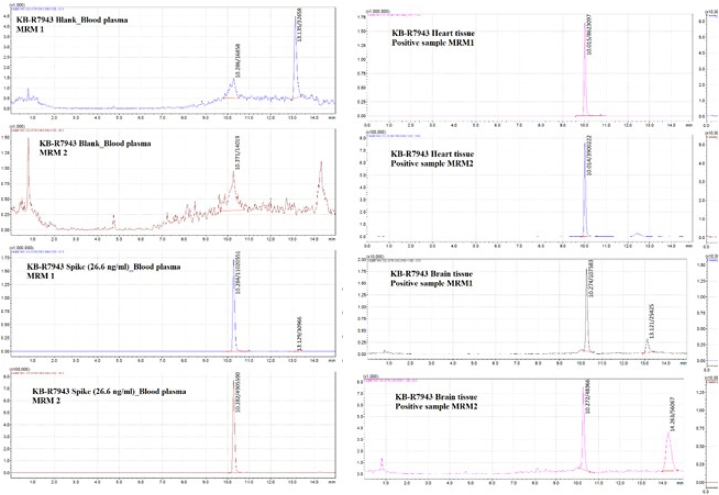
Left panel: MRM chromatograms: blank and spiked with 26.6 ng/ml KB-R7943 samples of rat blood plasma. Right panel: MRM chromatograms of KB-R7943 in positive samples of different biomatrices. (LCMS-8050, Shimadzu).

Two product formation reactions were used for analyte identification (MRM transitions), and one reaction for quantitation (Fig. 2).

#### 2.4.1 Bio-samples

200 mg of the sample (plasma, urine, homogenate) was transferred into 1.5 ml Eppendorf tubes, 10 μl of the internal standard solution was added and thoroughly mixed using a rotary shaker, and then 1 ml of the extracting solution of 0.1% formic acid in acetonitrile, was added. After ultrasonic stirring for 15 min, the tubes were centrifuged at 14,000 rpm for 5 min. Approximately 1 ml of the supernatant was decanted and transferred to a 15 ml Falcon plastic tube, 1 ml of the extracting solution (0.1% formic acid in acetonitrite) was added to the precipitate, and the extraction procedure was repeated. Three successive extracts were combined, 3 ml of hexane was added, and mixed on a rotary shaker for 5 min, after which the top layer of hexane was separated, and the purification procedure was repeated 2 more times. The purified extract was transferred to a 5 ml glass vial and evaporated to dryness in an FMS Super Vap 24 at 45 °C under nitrogen flow. 200 μL of the extractant was added to the vials, and the remains of the analyte were washed off the walls of the vial by stirring the solution on a rotary shaker for 3 min, after which the resulting solution was transferred into glass vials for HPLC analysis. With each batch of samples, appropriate matrix controls were prepared and extracted with the addition of KB- R7943 at 0, 0.1, 1, 10, 100, 1000, 5000, 20,000, and 200,000 ng/g and extracted simultaneously with the samples (Fig. 2).

#### 2.4.2 Validation

Analyte-free matrices were spiked to obtain the following concentrations: at 0, 0.1, 1, 10, 100, 1000, 5000, 20,000 and 200,000 ng/g for KB-R7943 to validate this method. Six replicates were employed for each concentration, and the experiment was repeated on three different days. The limit of quantification (LOQ) is defined as the lowest concentration of compounds in a matrix sample that gives a signal-to-noise ratio greater than 10 at a secondary MRM transition. For all matrices, the LOQ was 0.1 ng/g for KB-R7943.

The linearity of the method was evaluated using calibration curves constructed using matrix samples (spikes) with the addition of analytes. A correlation coefficient greater than 0.95 is an acceptable value for linearity; the closer the value is to one, the better the method is linear for the analyte. According to the European regulation 2021/808, the precision of a method, i.e. the closeness of a match between an acceptable true reference values, is determined by correctness and precision. In the absence of certified reference materials, the correctness and accuracy of measurements should be determined by experiments using spikes with a known concentration of analytes applied. The achieved confidence in recovery, repeatability, and within-laboratory reproducibility (RSDr) is shown in Table 2. Three samples were used to calculate RSD in the presented method. The results obtained during the validation cannot be compared with a similar method since no other methods for analyzing KB-R7943 in similar matrices were found.

**Table 2:** Coefficient of determination (R^2^), trueness, precision under repeatability (RSDr) conditions, and matrix factor of KB-R7943.

Matrix	R^2^	Lower limit of linearity range, (ng/g)	Upper limit of linearity range, (ng/g)	Trueness (%)	Conc^a^ (ng/g)	RSDr (%)	Mean Matrix Factor	Matrix Factor RSD (%)
Brain	0.970	1.0	1000	102.5	266	14.7	1.03	17.2
Heart	0.994	50.0	10,000	96.1	266	16.0	0.97	14.6
Blood plasma	0.999	0.1	200	97.4	266	3.2	0.98	4.2
Urine	0.998	1.0	1000	98.1	266	5.1	0.98	4.4

a Concentration for which RSDr was calculated.

The matrix effect is the effect a matrix can have on calculating an analyte concentration. It is evaluated by comparing the instrument's response to compounds dissolved in pure extractant with a sample corresponding to the matrix extract. The matrix factor (MF) for each analyte was calculated as the peak area of the standard corresponding to the matrix compared to the peak area of the standard solution. The relative standard deviation MF, calculated as the average MF value obtained for the concentration range from LOD to 2000 ng/g, was less than 20% in all cases, which is a satisfactory value per the European Regulation 2021/808 (Table 2).

### 2.5 Design of the in vivo experiments

#### 2.5.1 Animals

For both Experiment 1 and 2, male Wistar rats, 10–12 weeks old, were used. Water and standard laboratory chaw were supplied ad libitum. In Experiment 1, animals were housed individually in metabolic cages, whereas rats from Experiment 2 were placed in a cage of three animals. A constant room temperature of 22 °C was maintained during the experiments. All efforts were made to minimize animal suffering. Experiment 1 was held in the Sechenov Institute of Evolutionary Physiology and Biochemistry of the Russian Academy of Sciences (IEPhB RAS). It aimed to conduct pilot pharmacokinetic study of the orally applied KB-R7943. For Experiment 2, rats were from the Vivarium of the Medical University of Sofia. The aim was to perform neurobiological testing.

#### 2.5.2 Diabetes model

On the day of diabetic state induction, rats were deprived of food for 6 h (from 8 a.m. to 2 p.m.). A single i.p. injection was applied with 55 mg/kg STZ in the ice-cold citrate buffer (0.1 M pH 4.4) [31]. The observed polydipsia, polyuria, reduced thriving with lower body weight, and poor coating appearance indirectly indicated the development of diabetes. On day 28, after 6 h of fasting, blood glucose was measured with an Xpress Statstrip Glucometer (Nova Biomedical Corporation, Waltham, MA, USA) from the tail vein. A cut-off value of blood glucose >13 mmol/l was adopted. Those animals who did not meet the glucose cut-off value were reinjected and reassessed. Controls were once i.p. injected with ice-cold citrate buffer 0.1 M pH 4.4 only.

#### 2.5.3 General design considerations

Reportedly, amitriptyline 10 mg/kg daily reduces depression-like behavior and neuropathic pain and mitigates cognitive impairment in rats [32]. Decreased seizure susceptibility was reported after KB-R7943 pretreatment with a dose of 10 mg/kg [33]. So, it was decided to test the KB-R7943 in two doses, i.e., 10 mg/kg and half this.

#### 2.5.4 Group size considerations

Experiment 1 was designed as a pilot study. No sample size calculation was done in advance.

Experiment 2 includes sample sizes recommended in the corresponding protocol for STZ-induced diabetic models in mice and rats, where the recommended group size is from 12 to 20 [34]. No power analysis was performed in advance. In both experiments, animals were randomly grouped and allocated by a person from the laboratory staff not participating and not familiar with the investigations.

#### 2.5.5 Experiment 1

**Fig. 3:**
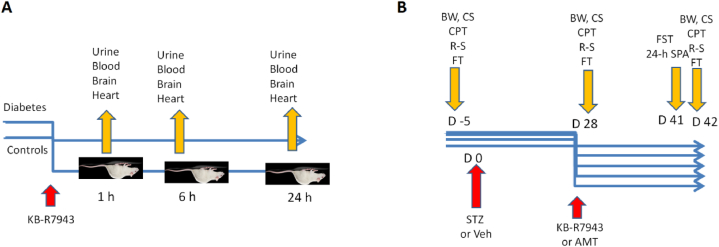
Design of the study. Panel A – Experiment 1. Panel B – experiment 2. BW – body weight; CS – coat status; CPT – cold plate test; R–S – Randall-Selitto test; FT – formalin test; FST – forced swim test; 24-h SPA – 24-h spontaneous physical activity; D – day.

Experiment 1 (Fig. 3A) was designed as a pilot PK study to assess the body distribution of orally applied KB-R7943 and to inform about some important PK parameters. Two groups, i.e., controls (n = 9) and STZ-induced diabetic models (n = 9), were included in the experiment. Each rat was placed in an individual metabolic cage and kept there until the administration of the drug. The KB-R7943 was applied once, p.o., in a 10 mg/kg dose with gavage. OraPlus was a vehiculum (16 gauge feeding tube 10 ml/kg). After applying of the KB-R7943, each rat was returned to their cage, and blood and urine samples were collected at 1st, 6th, and 24th hours (different n = 3 at each time-point). Metabolic cages were specially designed to separate feces from urine through the funnel, and a separation cone was used.

Blood samples were collected in EDTA-containing tubes with cardiopunction under ketamine-xylasine anesthesia (100 mg/kg and 10 mg/kg, respectively). After 1 h incubation (+4/+8 °C), blood was centrifugated 1000 *g*, and the plasma was used for the analysis after that.

Isolation of tissue samples of the heart and parts of the brain was performed after perfusion of the upper body of the rat with a phosphate-buffered saline (PBS) using a perfusion pump (BT100-2J LongerPump, Halma PLC, UK). Homogenates were prepared as 1:4 ratios (v/v) with PBS and stored at −80 °C until analysis. Three parallel samples of 0.03–0.06 g were prepared from each sample for quantitation.

#### 2.5.6 Pilot PK study

Pilot PK analysis was conducted with the freely available PK calculator at https://dash.gallery/dash-pk-calc/. The median values from the three measured concentrations per time point from both control and diabetic groups were used for deriving PK parameters for the assessment, namely biological half-lives (T1/2), area under curves (AUC _0-24_, AUC _0-inf_.), % extrapolation, maximal concentration (Cmax) and Tmax (time to reach the maximal concentration).

Clearances were calculated as dose (10 mg/b.w. for a rat weight 310 g average in the control group and 280 g in the group with diabetes) divided by AUC_0-24_. The volume of distribution was calculated as dose/(λ × AUC _0–24_). The terminal slope was calculated from the linear regression models after the ln transformation of the concentration values. Tissue penetration ratio was calculated separately for controls and diabetic rats as AUC_cortex_/AUC-_plasma_, AUC-_hypothalamus_/AUC-_plasma_, AUC-_hippocampus_/AUC-_plasma_, and AUC-_heart_/AUC-_plasma_, and urine penetration as AUC-_urine_/AUC-_plasma_.

#### 2.5.7 Experiment 2 – neuropharmacological assessments

Three animals per cage were housed for the experiment 2 (Fig. 3B). Rats were gently manipulated daily before undertaking the actual behavior tests. One week after adaptation, the Randl-Stellito (R–S) test, cold plate test (CPT), and formalin tests (FT) were performed at baseline. After that, the diabetes model was made with 48 rats. Twelve animals served as controls. Body weight (BW), coat score (CS), CPT, R–S, and FT were evaluated on day 28th post-STZ, and after that, rats were distributed into the treatment groups as follows.

• C-Veh – Controls, vehiculum treatment (n = 12)
• NP-Veh – STZ-treated rats with neuropathy, vehiculum treatment (n = 12)
• NP-KB-R7943 – 5 mg - STZ-treated rats, with neuropathy, KB-R7943 5 mg/kg treatment (n = 12)
• NP-KB-R7943 – 10 mg – STZ-treated rats, with neuropathy, KB-R7943 10 mg/kg (n = 12)
• NP-AMT – 10 mg - STZ-treated rats, with neuropathy, amitriptyline 10 mg/kg (n = 12).

OraPlus was used as a vehiculum for gavage (16 gauge feeding tube 10 ml/kg) in all rats. KB-R7943 in two doses (5 or 10 mg/kg) or amitriptyline 10 mg/kg was applied once daily until day 49. Behavior tests were performed 12 h after the oral gavage of the corresponding day, in the afternoon, starting from 2 p.m. No one rat died after establishing the DN model and during the amitriptyline or KB-R7943 treatment. On day 41, an FST was performed, and at 2 p.m., rats were placed individually for 24 h to assess spontaneous physical activity. After that, on day 42, the same battery of the tests as on day 28 were reassessed.

### 2.6 Coat state, water consumption, and body weight

Coat state, water consumption, and body weight were evaluated weakly. Eight body parts were considered separately: head, neck, dorsal coat, tail, forelimb, hind limb, ventral coat, and genital region [35]. The total score of the coat status was obtained by attributing a score of 0 (clear coat) or 1 (dirty coat or in the abnormal state) to each of the eight parts. Water consumption was measured per cage. The BW was measured individually and weekly.

### 2.7 Cold plate test

This test was used for allodynia testing. The rats were placed in a transparent cylinder. The plate (Ugo Basile, Gemonio, Italy) was set at 5 °C [36]. Two independent observers recorded the time-to-paw lifting. The cut-off time was set at 60 s. The maximum possible effect was derived as follows:

$$\%\text{MPE}=\frac{Reaction\;time\;after\;treatment\;–\;Control\;reaction\;time}{60\;\sec\;–\;Control\;reaction\;time\;}x\;100$$

### 2.8 Paw pressure test

The Randall-Selitto test detects mechanically induced pain with relatively considerable variability [37]. Before the test, each animal was carefully handled; then, it was placed into a soft cotton cloth and carefully immobilized with the same hand used to hold the tested paw. A Paw pressure test was performed 1 h after the cold test, allowing acclimatization to room temperature. The test was performed in triplicate for each animal on the same day at least half an hour apart. Paw mechanical sensitivity was tested with the analgesia meter (Ugo Basile, Gemonio, Italy). A constantly increased force rate (32 g/s) was applied to a punctiform area on the dorsal surface of the right hind paws and a reaction upon a corresponding gram pressure was recorded. No more than 150 g was applied to avoid paw damage. Accordingly, data were normalized as follows:

$$\%\text{MPE}=\frac{Reaction\;gram\;after\;treatment\;–\;Control\;reaction\;gram}{150\;g\;–\;Control\;reaction\;gram}\;x\;100$$

### 2.9 Formalin test (FT)

A low concentration of formalin is suitable for revealing allodynia in rats with diabetic neuropathy [38]. The formalin test was performed 1 h after the R–S test to decrease the interaction with the mechanical stimulus from the previous test. For the same reason, the left hind paws were injected. A needle (U-100 Insulin Syringe, Beckton and Dickinson, Dublin, Ireland) was used. A low-dose intraplantar injection of formalin 50 mcl 0.5% was done, and rats were placed in individual transparent cases for observation. Two independent observers measured the time spent licking or biting the injected paw, and the first (0–5 min) and the second post-injection phase (20–30 min) were reported in sec.

### 2.10 Forced swim test (FST)

The FST is often used to assess rodent depressive-like behavior [39]. More precisely, it measures the stress-coping strategy of rodents placed in an inescapable water container [40]. After the first active phase of climbing or swimming, a despair-like phase (floating and drowning) follows. A 15-min pre-test was conducted a day before the test. Rats were placed in a transparent Plexiglas tank (diameter 25 cm, height 60 cm) filled with 30 cm tape water. Immobility time was recorded in sec from a total 5-min test by two researchers, unaware of the treatment status of the rats. The water was changed for each rat. After removal from the tank, the rat was dried with a towel and temporally placed in a dried cage for observation.

### 2.11 24-Hour spontaneous physical activity

At the end of the trial, on day 41 at 2 p.m., rats were individually housed in cages (26 × 43 × 19 cm) equipped with infrared movement detectors connected to a computer. Three 16-infrared beam arrays, one on the longer site next to the floor and two on the short side of the rectangle, one next to the floor and a second at 5 cm above (for detecting vertical movements), were used [41]. Each cross of the infrared array by the animal generated a signal (beam break), which was recorded by the computer. The 24-h signal sums evaluated after that [42].

### 2.12 Recovery with intervention (% of maximum)

The effectiveness of the long-term intervention at improving behavior parameters was calculated as follows [43]:

$$\left[\begin{array}{c} Abs\;\left(NPtreated-meanNPveh\right)\\ \_\\ Abs\;\left(meanNPveh-mean\;Cveh\right) \end{array}\right]\;x\;100$$

### 2.13 Statistics

**Experiment 1**. For better visualization of the PKs and due to the low sample numbers, KB-R8943 concentration data from 3 animals per group per time point were presented with their median values. After the ln transformation of the concentration values, simple linear regression models were built to quantify the relationship between time and plasma concentration in plasma, heart, cortex, hypothalamus, hippocampus, and urine separately for controls and diabetic rats.

**Experiment 2.** Data were checked for normality of distribution (Shapiro-Wilk's test) and equality of variances (Levene's test). A two-way repeated measure analysis of variances (RM-ANOVA) was applied for repeatedly measured variables. Both factors, Day (of the evaluation) and Group (to which the animal was assigned) were checked for interaction. For once-measured variables, parametric one-way ANOVA has been applied accordingly, followed by the Tukey post hoc test. SigmaPlot 11.0 and MedCalc v.20.111 were used for statistics and graphs.

## 3 Results

### 3.1 In silico characteristics

Promising bioavailability (F_30%_ or higher) with a probable brain-barrier penetration was predicted by ADMETlab 2.0. A relatively few toxicity outcomes are expected, albeit both used online platforms issued mutagenicity and stress response related to mitochondrial membrane potential influence. ProTox-II predicted the LD_50_ 500 mg/kg (relative to toxicity class 4) with 54.26% prediction accuracy. Possible drug-induced liver injury (DILI), carcinogenicity, skin sensitization, respiratory toxicity, aromatase, and estrogen receptor activity were additionally predicted from ADMETlab 2.0.

### 3.2 Pilot PK assessment of KB-R7943 in controls and STZ-induced diabetes in rats

**Fig. 4:**
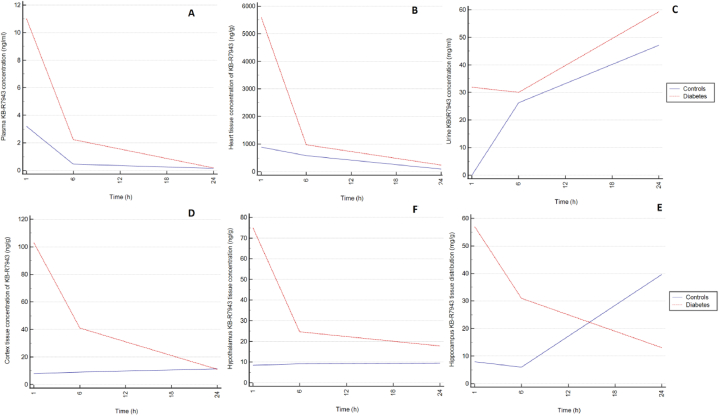
Tissue distribution of orally applied KB-R7943 10 mg/kg in rats with diabetes (red lines) and controls (blue lines). Panel A – plasma. Panel B – heart. Panel C – urine. Panel D – cortex. Panel E − hypothalamus. Panel F – hippocampus. Data are logarithmically transformed and are presented as median value and ranges from 3 rats per group per time point. (For interpretation of the references to colour in this figure legend, the reader is referred to the Web version of this article.)

After oral application, KB-R7943 entered the systemic circulation with a Tmax of 1 h. The plasma level of KB-R7943 thereafter decreased for 24 h. For the same time, the level of the unmetabolized drug in the urine progressively increased. KB-R7943 penetrated in the heart and brain tissues (cortex, hypothalamus, and hippocampus), where KB-R7943 remained at detectable levels for at least 24 h. This warns to expect a possible activity from both neuronal structures and the heart – Fig. 4.

Over 3 times higher AUC_(0-24)_ (60.60 versus 16.40 ng/ml*hr), over 7 times lower Vd (288.78 vs. 2100.27 L), over 4 times lower systemic clearance (46.20 vs. 189.02 L/h), and over one and half times faster decline of the plasma concentration (−0.16 vs. −0.09) in rats with diabetes vs. controls were observed. The heart and hippocampus KB-R7943 penetration in the control group was 1.2 times higher (621.80 vs. 499.39) and over 2.5 times higher vs. diabetic rats respectively – Table 3. The small number of animals per group does not allow performing statistical assessment.

**Table 3:** Pharmacokinetic parameters of KB-R7943 in controls and STZ-induced diabetic rats. BW – body weight; T1/2 - biological half-lives; AUC- area under curves concentrations to time (AUC_0-24_, AUC_0-inf_); %Extrap - % extrapolation; Cmax maximal concentration; Tmax - time to reach the maximal concentration; ƛ – terminal slope; Cl – clearance; Vd – volume of distribution.

	Plasma		Cortex		Hypothalamus		Hippocampus		Heart		Urine	
	Cont	Diab	Cont	Diab	Contr	Diab	Contr	Diab	Contr	Diab	Contr	Diab
BW (g)	310	280										
T1/2 (hr)	6,10	4,20	−47,30	7,70	−1520	13,70	−8,80	11,60	7,00	5,70	NA	−23,20
AUC_(0-24)_ (ng/ml*hr)	16,40	60,60	232,40	880,40	215,40	668,10	449,40	645,40	10,197,50	30,257,00	727,20	974,40
AUC_(0-24)_ tissue^a^/AUC _(0-24)_ plasma			14.17	14.53	13.13	11.02	27.40	10.65	621.80	499.39	44.34	16.08
AUC (0-inf) (ng/ml*hr)	17,70	61,70	−552,90	1003,20	−1867,30	1019,50	−52,20	865,00	11,134,40	32,270,20	NA	−1017,40
%Extrap	7,40	1,90	142,00	12,20	111,50	34,50	960,80	25,40	8,4	6,20	NA	196,30
Cmax (ng/ml)	3,20	11,0	11,50	103,0	9,50	75,00	39,60	57,00	893,00	5600,00	47,20	59,30
Tmax (hr)	1,00	1,00	24,00	1,00	24,00	1,00	24,00	1,00	1,00	1,00	24,00	24,00
ƛ	−0,09	−0.16	0.02	−0.07	0.01	−0.04	0.04	−0.05	−0.11	−0.12	0.07	−0.04
Cl (L/hr)	189.02	46.20										
Vd (L)	2100.27	288.78										
QIVIVE -prediction F(up)	0,89											
Partitution coefficient			Brain 35,63						Heart 14,96		Kidney 17,44	

a Or urine.

One plasma sample from the control group at the 1st hour, two urine samples from the control group at the 1st hour, and one urine sample from the diabetes group at the 1st hour were with undetectable KB-R7943 concentrations. Simple linear regression was used to test if time significantly predicted drug concentrations in plasma, cortex, hypothalamus, hippocampus, heart and urine separately for controls and diabetic rats – Table 4.

**Table 4:** Simple linear regression models of the KB-R7943 concentrations as a function of the time.

Response variable	Regression model	Model assessment	
Plasma concentration - controls	Ln(Conc) = 0.423–(0.094*time)	F(1, 6) = 7.238, p = 0.036	β = 0.094, p = 0.036
Plasma concentration - diabetes	Ln(Conc) = 1.983-(0.163*time)	F(1,7) = 9.649, p = 0.017	β = 0.163, p = 0.017
Cortex concentration - controls	Ln(Conc) = 2.144+(0.015*time)	F(1,7) = 0.158, p = 0.703	β = 0.015, p = 0.703
Cortex concentration - diabetes	Ln(Conc) = 4.133-(0.075*time)	F(1,7) = 13.251, p = 0. 008	β = −0.075, p = 0.008
Hypothalamus concentration - controls	Ln(Conc) = 2.256+(0.007*time)	F(1,7) = 0.049, p = 0. 830	β = −0.007, p = 0.830
Hypothalamus concentration - diabetes	Ln(Conc) = 3.940-(0.045*time)	F(1,7) = 3.001, p = 0. 127	β = −0.045, p = 0.127
Hippocampus concentration - controls	Ln(Conc) = 2.073+(0.037*time)	F(1,7) = 1.351, p = 0.283	β = −0.037, p = 0.283
Hippocampus concentration - diabetes	Ln(Conc) = 3.845-(0.048*time)	F(1,7) = 3.681, p = 0.097	β = −0.048, p = 0.097
Heart concentration - controls	Ln(Conc) = 7.150-(0.107*time)	F(1,7) = 19.634, p = 0.003	β = −0.107, p = 0.003
Heart concentration - diabetes	Ln(Conc) = 8.414-(0.125*time)	F(1,7) = 7.987, p = 0.026	β = −0.125, p = 0.026
Urine concentration - controls	Ln(Conc) = 1.974+(0.074*time)	F(1,5) = 2.745, p = 0.158	β = −0.074, p = 0.158
Urine concentration - diabetes	Ln(Conc) = 4.264-(0.041*time)	F(1,6) = 0.639, p = 0.455	β = −0.041, p = 0.455

### 3.3 Body weight (BW) and coat state

**Fig. 5:**
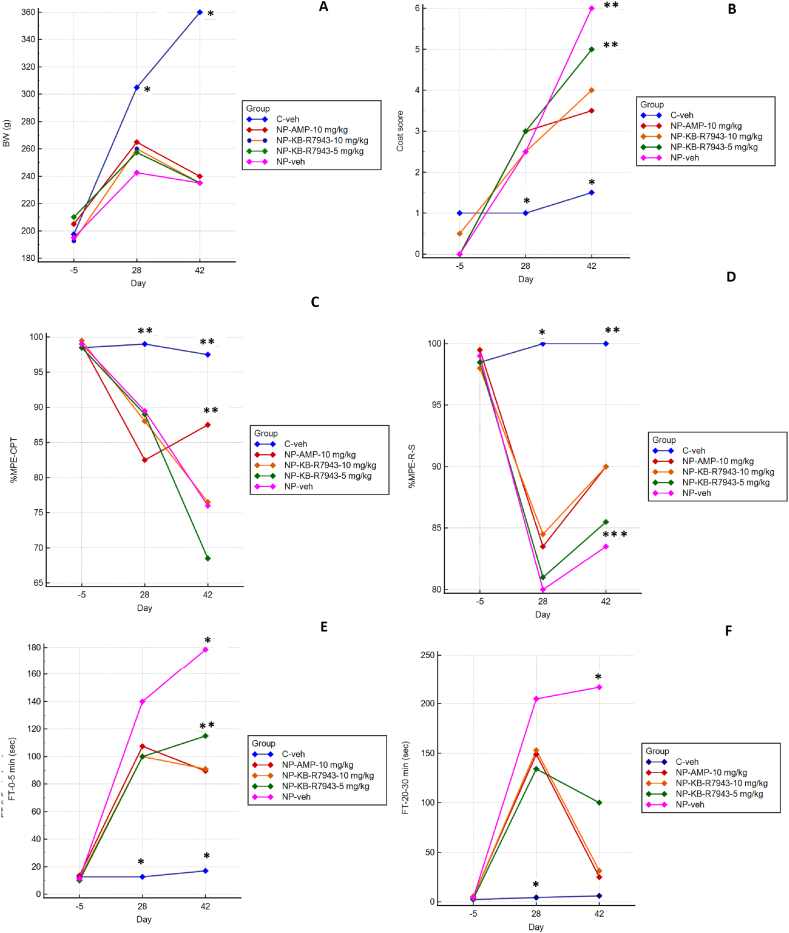
Chronic treatment effects. Data are presented as Least Square means. Panel A. Body weight (BW). Panel B. Coat score. Panel C. Cold plate test. Panel D. Randall-Stellito test. Panel E. Formalin test 0–5 min. Panel F. Formalin test 20–30 min (sec.). 2-way RM-ANOVA, Tukey post hoc, n = 12 in each group * p < 0.001 **p < 0.01 ***p < 0.05.

For the BW, a significant interaction between factors Day and Group was found with F (8,110) = 21.84, p < 0.001. The significantly higher body weight on day 28 (p < 0.001) and even more on day 42 (p < 0.001) was demonstrated for Cveh than all other groups. Differently treated diabetic groups did not differ statistically by BW (Fig. 5A).

The coat score also had a statistically significant interaction between factors Group and Day F(8, 110) = 15.77, P < 0.001. A poorer coating score was seen for all diabetic groups vs. Cveh P < 0.001, which was ameliorated under amitriptyline or KB-R7943 treatment (Fig. 5B).

### 3.4 Cold plate test

For data of the cold plate test, a significant interaction was found between factors Day and Group F (8, 110) = 9.67, p < 0.001. The positive cold plate test is thought to be indicative of allodynia [36]. Cold allodynia was demonstrated for all STZ-treated rats before the application of tested substances (p < 0.01 for Cveh vs. all other groups on day 28). At the end of the trial, only amitriptyline treatment demonstrated a reversal effect (p < 0.01 vs. all other diabetic groups) (Fig. 5C).

### 3.5 Paw pressure test

In the R–S test, there was a significant interaction between factors Day and Group F (8, 110) = 3.70, p < 0.001. The test was able to reveal the development of the mechanical allodynia, which was partly reversed by amitriptyline 10 mg (p < 0.05 for NP-AMP-10 mg/kg vs. NPveh) and to a comparable extent by KB-R7943 10 mg/kg (p < 0.05 for NP-KB-R7943-10 mg/kg vs. NPveh). (Fig. 5D).

### 3.6 Formalin test (FT)

The two phases of the formalin test were analyzed separately. A low concentration of formalin practically did not provoke an antinociceptive reaction (licking, guarding, or flinching of the afflicted limb) in the control group, in contrast to the diabetic neuropathy groups (p < 0.001 for Cveh vs. all other groups from the same time point). An intensified behavior reaction was observed between 0 and 5 and between 20 and 30 min of the test for all rats with DN.

For the first phase, there was a significant interaction between factors Group and Day with F (8, 110) = 11.49, p < 0.001. Both amitriptyline and KB-R7943 10 mg/kg attenuated this antinociceptive-like behavior in the first 5 min of the formalin test (p < 0.01 for NP-AMP 10 mg/kg and NP-KB-R7943 10 mg/kg vs. NP-Veh) (Fig. 5E). The lower dose of KB-R7943 produced a weaker effect (p < 0.01 for NP-KB-R7943 5 mg/kg vs. NP- KB-R7943 10 mg/kg and NP-AMP 10 mg/kg).

For the second phase of the formalin test, an interaction between factors Day and Group was found, F (8,110) = 24.42, p < 0.001. As with the first phase of the test, the formalin injection practically did not produce an antalgic behavior reaction in control animals. Conversely, in all diabetic groups, a significant increase in antinociceptive behavior was noticed on day 28 (p < 0.001 for Cveh vs. all other groups). This effect was attenuated both by amitriptyline and KB-R7943 (p < 0.001 for NPveh vs. all other groups from the same time point) in a dose-dependent manner (p < 0.001 for NP-KB-R7943 5 mg/kg vs. NP- KB-R7943 10 mg/kg and NP-AMP 10 mg/kg from the same time point) (Fig. 5F).

### 3.7 Forced swim test (FST)

**Fig. 6:**
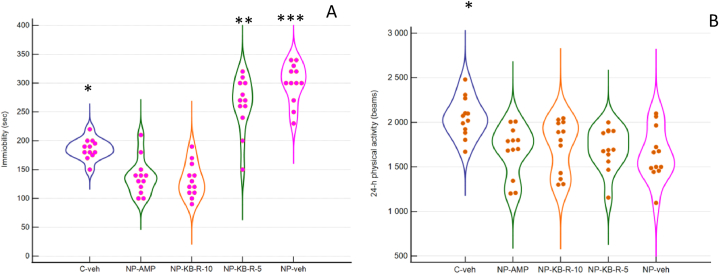
Panel A. Data distribution of the immobility time in FST. Panel B. Data distribution of the 24-h spontaneous physical activity. One-way ANOVA, Tukey post hoc test, n = 12 in each group.*p < 0.05 for Cveh vs. all other groups; **p < 0.001 for NP-KB-R7943 5 mg vs. NP-KB-R7943 10 mg and NP-AMP 10 mg; ***p < 0.001 for NPveh vs. NP-KB-R7943 10 mg and NP-AMP 10 mg.

One-way ANOVA was applied to compare the effect of the allocated group to the immobility time in FST. A significant effect was demonstrated with F (4, 55) = 59.04 p < 0.001, and the immobility time of rats from the Cveh group (185.83 ± 17.69 s) differed significantly from all other groups (p < 0.05). NPveh group (300.00 ± 34.64 s) demonstrated significantly longer immobility time vs. amitriptyline treated (137.50 ± 31.94 s), p < 0.001, as well as vs. the higher dose of 10 mg of KB-R7943 (131.67 ± 29.80 (p < 0.001) (Fig. 6A).

### 3.8 24-Hour spontaneous physical activity

One-way ANOVA was applied to compare the effect of the allocated group to the 24-h physical activity. A significant effect was demonstrated with F (4, 55) = 4.561 p = 0,003. Rats from the C-veh group had significantly higher 24-h activity (2051.58 ± 225.34 beams) than all groups with diabetic neuropathy, irrespectively of whether they received or no treatment, i.e., 1639.67 ± 292.78 beam breaks for NPveh (p < 0.05); 1678.33 ± 282.00 beam breaks for NP-AMP = 10 mg/kg (p < 0.05); 1705.92 ± 237.74 beam breaks for NP-KB-R-5 mg (p < 0.05), and 1733.08 ± 297.65 beam breaks (p < 0.05). On the other hand, no difference was found among differently treated groups (Fig. 6B).

### 3.9 Comparison of the treatment effects

**Fig. 7:**
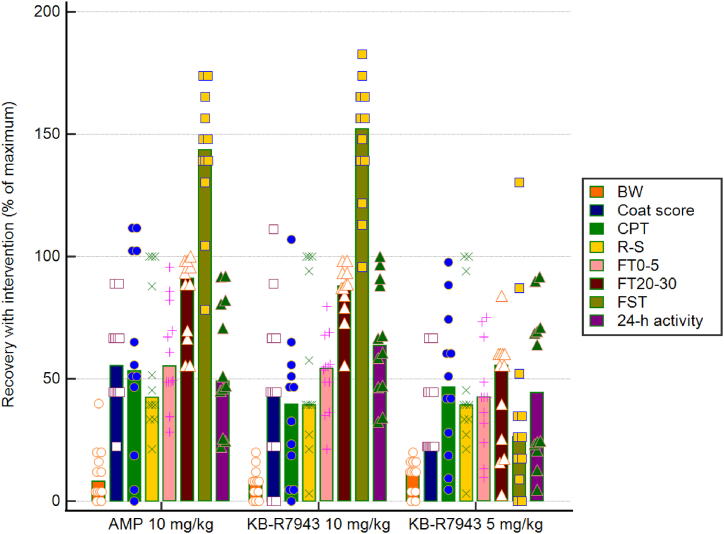
Behavior effects of the chronic treatment with KB-R7943. Data are represented as means and individual values, calculated by the formula in paragraph 2.12 of the text. There was no statistical analysis of this data set because there are no a priori reasons to identify what level of recovery is necessarily biologically significant clearly. BW- body weight; CPT-cold plate test; R–S – Randall- Stelitto test; FT0-5 – formalin test (0–5 min); FT20-30 formalin test (20–30 min); FST – forced swim test.

Dose-dependent behavior effects of the chronic oral treatment with KB-R7943 were compared vs. amitriptyline 10 mg/kg orally (Fig. 7). KB-R7943 at a dose of 10 mg/kg had a similar pharmacological profile to the amitriptyline 10 mg/kg in rats given the used tests. The highest effect of the treatment was noticed in the second phase of the formalin test.

## 4 Discussion

To our understanding, this is the first study to compare PK of orally applied KB-R7943 in the model of diabetes vs. control rats. It is also one of the few studies evaluating the effect of orally applied KB-R7943 in a DN model in rodents. A relatively quick heart and brain penetration of the tested drug in both studied groups was revealed, as well as a tendency to remain in those tissues in detectable concentration for a minimum of 24 h. Urinary excretion of the unmetabolized drug started within the 1st hour, and urine concentration increased with time. Higher drug exposure (AUC), lower volume of distribution (Vd) and clearance (Cl), and faster decline of the plasma concentration (ƛ) in rats with diabetes vs. controls were observed. The heart and hippocampus KB-R7943 penetration (AUC _tissue_/AUC _plasma_) was higher in controls vs. diabetic rats.

Relatively higher drug exposure in diabetes rats can be due to gastrointestinal stasis, a typical complication of diabetic neuropathy. Longer stays in the gastrointestinal tract will increase the amount of the absorbed drug. However, we did not directly measure gastrointestinal movement in this study. Another plausible factor for the higher AUC in diabetes could be changes in the plasma protein binding, probably due to changes in intestinal hormones (incretins) or posttranslational protein modifications under high blood glucose (non-enzymatic protein glycation). For the KB-R7943, the QIVIVE predicts 89 % plasma protein binding, and due to the acidic nature of KB-R7943, we can presume that it binds preferentially with the plasma albumin [44]. Protein glycation changes the binding capacity of plasma proteins and can cause an increase in the KB-R7943 unbound fraction.

Plasma protein binding is essential for hepatic and renal clearance, tissue distribution, and pharmacological effects. Here, we experimentally demonstrate that KB-R7943 successfully enters into the brain and remains there for at least 24 h. As other investigators showed, STZ-induced diabetes progressively increases the BBB permeability to small molecules due to microvascular damage [45]. On the other hand, KB-R7943 markedly prolongs osmotically initiated blood-brain barrier opening without causing brain damage [46]. This effect was attributed to the regulation of Ca2+-mediated thigh junction opening by KB-R7943 [47] and is probably responsible for the KB-R7943 central penetration.

When we discuss drug distribution, two additional factors should be considered: 1) diffusion of the free drug depending on the pH gradient of the plasma versus the intracellular space, and 2) Albumin-mediated uptake phenomenon. Although these two factors are discussed with the hepatic distribution of drugs [48], we propose they could also be applied to other organs, e.g., the brain and kidneys. Here, we report higher hippocampus and heart penetration of the KB-R7943 in controls vs. diabetes rats. Under normal physiological conditions, the extracellular pH is maintained at approximately 7.4, with the intracellular pH ranging between 7.2 and 7.4 [49]. Reportedly, the extracellular pH decreases under hypoxic and ischemic conditions; for example, during experimental focal ischemia, brain pH drops to 6.0 [50]. We did not measure cellular pH or brain or heart circulation; however, in light of the known vascular damage in diabetes, we can suspect similar processes in our diabetes model.

In the case of the albumin-mediated uptake phenomenon, the transporters involved in implementing barrier functions in the body are essential. The phenomenon of albumin-mediated hepatic uptake of organic-inorganic anion transporter (OAT) substrates has recently been demonstrated [51]. However, OATs are expressed in virtually all barrier systems in the body.

Moreover, because our investigated drug candidate KB-R7943 is a weak acid at physiological pH, we assume it could also accumulate in organs using these transporters. OATs protect the body from xenobiotics and serve as remote sensors and signal transmissions for organogenesis and organ maturation, recovery from damage, communications across the placenta, and even between individual organisms by engaging in the olfactory function [52]. Moreover, known glucose-sensing properties of some OATs [53] could also be a factor for the different KB-R7943 distribution in diabetes vs. control.

Our findings of analgesic effects of the KB-R7943 are in line with those of other investigators reporting behavior improvement (von Fray and Hargreaves tests) after KB-R7943 intrathecal injection in a spinal nerve ligation model of neuropathic pain, demonstrating the importance of the central mechanism of action of KB-R7943 [54]. The model we used was confirmed with significantly higher glycemia, together with neurobehavior changes suggestive of the development of allodynia and depressive-like behavior. Allodynia (pain due to a stimulus that does not usually provoke pain) and hyperalgesia (increased pain from a stimulus that generally causes pain) are prominent neuropathic pain symptoms. The mechanistic basis of this enhanced sensitivity needs to be understood. Our study demonstrates an alleviation of cold mechanical and formalin-induced allodynia in a dose-dependent manner. No significant adverse effects or death attributable to KB-R7943 were observed in this trial. Our study also demonstrates partial reversal effects can be obtained with chronic oral treatment. The two doses of KB-R7943 we selected for testing were based on previous publications demonstrating the neuropharmacological activity of the orally applied KB-R7943 to suppress seizures induced by alcohol withdrawal [33] and pentylenetetrazole injection [55]. Higher doses are supposed to interact with other transporters and reduce the beneficial effect [56]. In practice, it is a common approach to test for analgesic effects of substances in doses demonstrating antiseizure activity.

Both central and peripheral effects of KB-R7943 can be anticipated based on NCX's known central and peripheral distribution. The flicker type of the movement of NCX in dependence on Na+ and Ca2+ in the microenvironment presents a potential to stabilize the movement mode in the desired direction with the application of reverse-mode inhibitors like KB-R7943. In our experiment, the highest effect was observed in the second phase of the formalin test, known to be due to central sensitization [57]. The peripheral neuronal mechanism of action of KB-R7943 is also anticipated. Other investigators reported the partial prevention of linezolid-induced sensory axonopathy by KB-R7943 [58]. The peripheral mechanism is involved in the first phase of the formalin test, which was also affected by the tested drug. As was demonstrated by other investigators, the NCX blockade with KB-R7943 partially protects neurites from injury due to energetic stress [59].

KB-R7543 is an isothiourea derivative inhibitor of Na-dependent Ca uptake. It inhibits the three existing sodium-calcium exchangers with higher affinity for NCX3 [60]. It flickers between forward- and reverse modes to coordinate the influx and efflux of Na+ and Ca2+ with significant electrophysiological consequences [61]. Recently, the leading role of NCX3 in pain “windup” referring to a progressively increased response of predominantly deep dorsal horn neurons upon repetitive nociceptive stimulation was reported. This phenomenon explains the temporal summation of the pain perception, which is known to be disturbed in DN. Reportedly, NCX3 knock-out mice demonstrate an increased pain-related behavior and spinal hyperexcitability, whereas an enhanced spinal expression of NCX3 reduced pain in mice [62]. Here, we have demonstrated the inverse mode NCX with the blocker a univocal effect. Numerous data indicate the primary role of glutamate ionotropic receptors N-methyl-d-aspartate (NMDAR) in the pathogenesis of neuropathic pain syndrome, while regulators of sodium-calcium exchange (NCX) play an essential role in the regulation of NMDAR ion conductivity. These regulators, indirectly through their action on the NMDA receptor, can participate in the processes of relieving pain [63].

With the introduction of positron emission tomography, it became possible to identify that depression can be a prodromal of neurodegeneration [64]. Depression itself is found to be two to three times as prevalent in people living with diabetes [65], and a strong correlation of hyperglycemia with neurodegeneration was found [66]. Recently, the fast-acting antidepressant activity of sub-anesthetic doses of ketamine was attributed to activating the L-type calcium channels, increasing Ca2+ intracellular entry, and enhancing the synthesis of the brain-derived neurotrophic factor (BDNF) [67]. Interestingly, a recently published systematic review and meta-analysis points to calcium channel blockers as a potential risk factor for depression [68]. Alleviation of neuronal degeneration was observed after nimodipine-induced direct (calcium channel blockade) and indirect (subsequently inducing NCX) decrease in cellular calcium level and oxidative stress [69]. In our experiment, the coat score demonstrates poor self-care behavior as a sign of depressive mood. The coat score is also dependent on the dysmetabolic state due to diabetes. On the other hand, the FST, traditionally discussed in terms of depressive-like behavior, in our experiment can also be attributed to neuropathy or the general decreased animals' well-being due to diabetes and the reduced animals' energy.

Amitriptyline, a long-time used for DN treatment, also demonstrated a voltage-gated sodium channel modulator activity, Ca-dependent NMDA receptor desensitization, and trapping channel block [28]. The active comparator in this study was amitriptyline, an old tricyclic antidepressant drug with multiple mechanisms of action, including blockade of the reuptake of both serotonin and norepinephrine, with a strong binding affinity for alpha-adrenergic, histamine (H1), and muscarinic (M1) receptors [70], voltage-gated sodium channel modulator activity and Ca-dependent NMDA receptor desensitization [28]. Its active metabolite nortriptyline further expands molecular targets. Some investigators affirmed the putative neurotrophic activity of amitriptyline [71], and others could not [72]. Reportedly, amitriptyline reduces acute [73] and chronic cold allodynia [74]. The inhibitory activity of amitriptyline on NCX [75] and the upregulation of NMDA receptors by NCX in physiological conditions were also reported [76] In the in vitro studies, the Ca2+-dependent NMDA desensitization by KB-R7943 was also demonstrated [77].

The 4-h spontaneous physical activity was significantly suppressed in all STZ-treated rats. It could be attributed either to the experienced discomfort due to the neuropathy or to the sedative effect of the treatment. Such an effect is well known for amitriptyline, whereas data about KB-R7946 are scarce. We cannot also exclude the development of depressive comorbidity in DN. The coexistence of depression and diabetes is twice as frequent as would be predicted by chance alone [78], and painful DN is the strongest predictor of depression [79], increasing its incidence by 24% [61]. A possible underlying common mechanistic link goes between oxidative stress and Ca2+ signaling, notably by activation of some Ca2+-permeable transient receptor potential (TRP) cation channels, which depend on cellular redox status. It is a common mechanism discussed both in psychiatric disorders, including depression, as well as in painful DN [80].

### 4.1 Limitations

Our study has several limitations. In Experiment 1, the small groups (n = 3) in three-time points are insufficient for a complete and more precise PK study. For the same reason, statistics is inapplicable. In Experiment 2, the neuropathy drug response biomarkers, other than behavior tests, were not measured. In both experiments, only one animal model was tested. The activity of NCX and other drug response targets was outside of this study's scope.

### 4.2 Conclusion

This study demonstrates the promising neuropharmacological activity of orally applied KB-R7943. The drug reaches its Tmax for approximately 1 h. It penetrates the heart and the brain in concentrations measurable in the 1st hour of the oral application, remaining in detectable levels for up to the 24th hour. KB-R7943 is eliminated in the urine, at least in part as unmetabolized. Those findings support that the heart, the brain, and the kidney are crucial organs for KB-R7943 exposure with therapeutic and side effects potential. Higher drug exposure, lower volume of distribution, and clearance and faster decline of the plasma concentration in rats with diabetes vs. controls were revealed. Higher heart and hippocampus penetration of the KB-R7943 in controls vs. diabetic rats was demonstrated. Although it is generally accepted that diabetes mellitus does influence the PK and pharmacodynamics (PD) of drugs, there is no valid general conception about the direction and involved mechanisms, and a recommendation is launched to study the PK and PD in diabetes drug-by-drug [81]. The advantage of oral application in chronic treatment is well recognized. From the toxicological point of view, cumulative tissue effects (brain, heart, and kidney) warrant further detailed characterization.

## Funding

Funding was received from the Bulgarian National Science Fund, Grant КП-06-Rusia 25/December 16, 2020, Grant KП-06-Н63/9 December 13, 2022; and State Reserves of IEPhB 10.13039/501100002674RAS, Russia № 075-00264-24-00. The funding sources had no involvement in study design; in the collection, analysis and interpretation of data; in the writing of the report; and in the decision to submit the article for publication.

## Ethics declarations statement

Approval was received from the IEPhB RAS Bioethics Committee (approval number: Protocol № March 2021 date: March 25, 2021) and from the Bulgarian Food Safety Agency (approval №330/2022), respectively.

## Data availability statement

Data are available by authors upon request.

## CRediT authorship contribution statement

**Pavlina Andreeva-Gateva:** Writing – review & editing, Writing – original draft, Visualization, Validation, Supervision, Software, Resources, Project administration, Methodology, Investigation, Funding acquisition, Formal analysis, Data curation, Conceptualization. **Milen Hristov:** Writing – review & editing, Visualization, Validation, Investigation, Formal analysis. **Margarita Strokova-Stoilova:** Writing – review & editing, Resources, Investigation, Formal analysis. **Natasha Ivanova:** Writing – review & editing, Methodology, Investigation, Formal analysis. **Zafer Sabit:** Writing – review & editing, Resources, Methodology, Investigation, Formal analysis, Conceptualization. **Slavina Surcheva:** Writing – review & editing, Validation, Data curation. **Mihail Beliakov:** Writing – review & editing, Writing – original draft, Methodology, Formal analysis, Data curation. **Georgi Karakashev:** Visualization, Methodology, Formal analysis, Data curation. **Ivan Sukhov:** Writing – review & editing, Methodology, Formal analysis, Data curation. **Daria Belinskaya:** Writing – review & editing, Methodology, Investigation, Data curation. **Natalia Shestakova:** Writing – review & editing, Resources, Project administration, Data curation, Conceptualization.

## Declaration of competing interest

The authors declare that they have no known competing financial interests or personal relationships that could have appeared to influence the work reported in this paper.
